# Supplementary material for: Conditional chemoconnectomics (cCCTomics) as a strategy for efficient and conditional targeting of chemical transmission
Source: eLife. 2024 Apr 30;12:RP91927. doi: 10.7554/eLife.91927 (PMC11060718; doi:10.7554/eLife.91927)
Supplement: Figure 3—source data 3. [file elife-91927-fig3-data3.zip › Figure 3 source data3 part 2.zip!/Figure 3-Source data 3-part2/1053-1-CRISPResso_Report_mpeqg9da.zip!/CRISPResso_on_1053_2/2b.Nucleotide_percentage_quilt_around_sgRNA_ggagcggaatctacaaaacg.pdf]

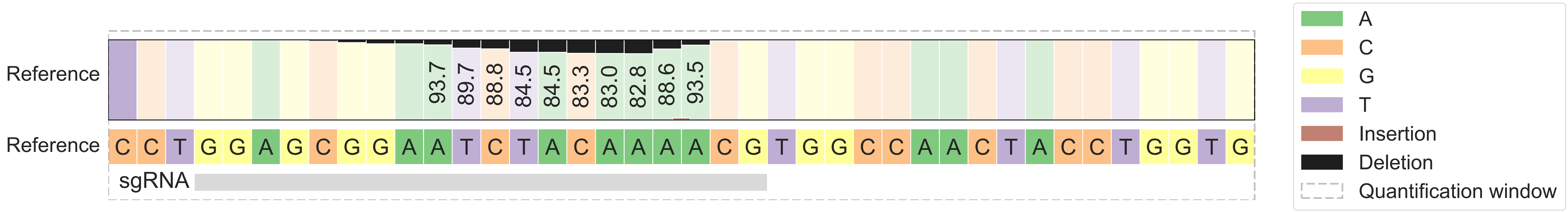
Reference
Reference
C C T G G A G C G G A A T C T A C A A A A C G T G G C C A A C T A C C T G G T G
sgRNA
93.7
89.7
88.8
84.5
84.5
83.3
83.0
82.8
88.6
93.5
A
C
G
T
Insertion
Deletion
Quantification window
